# Left atrial anatomical variations correlate with atrial fibrillation sources near the left atrial ridge

**DOI:** 10.3389/fcvm.2022.928384

**Published:** 2022-09-28

**Authors:** Rita B. Gagyi, Nándor Szegedi, Judit Simon, Sip Wijchers, Rohit Bhagwandien, Melissa H. Kong, Peter Ruppersberg, Tamas Szili-Torok

**Affiliations:** ^1^Department of Cardiology, Electrophysiology, Erasmus MC, Rotterdam, Netherlands; ^2^Heart and Vascular Center, The Semmelweis University Hospital, Budapest, Hungary; ^3^Ablacon Inc., Austin, TX, United States; ^4^Ablacon Inc., Blonay, Switzerland

**Keywords:** atrial fibillation, catheter ablation, electrographic flow mapping, left atrial appendage, left superior pulmonary vein

## Abstract

**Introduction:**

Anatomical variations and characteristics of the left atrium (LA) may have a previously undescribed effect on source locations in atrial fibrillation (AF). This is the first study aiming to investigate the relationship between anatomical characteristics of the LA and non-PV sources detected by electrographic flow (EGF) mapping in patients with persistent AF.

**Materials and methods:**

We analyzed cardiac computed tomography (CT) and EGF mapping data in patients who underwent radiofrequency catheter ablation (CA). EGF mapping is a novel method based on Horn–Schunk flow estimation algorithm, used to estimate cardiac action potential flow in the atria that can detect AF sources in patients with persistent AF. By analyzing EGF maps obtained during CA procedures, we localized non-PV sources in the LA.

**Results:**

Thirty patients were included in this study (mean age 62.4 ± 6.8 years). Ten patients had AF sources near the LA ridge, while twenty patients had no leading source (source activity > 26%) near the LA ridge. LA anatomical characteristics, left atrial appendage (LAA) length, and ostial diameter showed no correlation with the presence of a leading source. We documented 19 patients with abutting LAA and left superior pulmonary vein (LSPV) (distance < 2 mm), and 11 patients with non-abutting LAA–LSPV (distance > 2 mm). Three out of 19 patients presented with a leading source near ridge in the abutting LAA–LSPV group, while 7 out of 11 patients presented with a leading source near the ridge in the non-abutting LAA-LSPV group (*p* = 0.01).

**Conclusion:**

Our data suggests that non-abutting LAA-LSPV is associated with the presence of AF sources near the LA ridge.

## Introduction

Pulmonary vein isolation (PVI) provides a highly effective treatment option for patients with paroxysmal atrial fibrillation (PAF) (1). However, patients with persistent atrial fibrillation (AF) or long-standing persistent AF remain a challenging group to treat with PVI, owing to the long duration of the arrhythmia and pathophysiologic complexity that may include multiple triggers and abnormal substrate (2, 3). Long-term success rates after catheter ablation (CA) in persistent AF are significantly lower than in paroxysmal AF (2). This may be attributable to deficiencies in our understanding of underlying mechanisms of extra pulmonary vein (PV) sources. Research to date has not yet determined the exact relation of atrial substrate outside the PVs to the left atrial (LA) and right atrial (RA) anatomy. Previous studies show that the left atrial appendage (LAA) can represent a trigger site of AF, and may be responsible for arrhythmia recurrences in 27% of patients scheduled for redo CA (4). It has also been demonstrated that LAA isolation in addition to PVI may improve long-term outcomes in patients with persistent AF compared with the targeting PV alone strategy (5, 6). Anatomical variations of the LA may have an effect on source location in AF as in the case of paroxysmal AF patients. Electrographic flow (EGF) mapping is an innovative technique using computer vision and optical flow algorithm to estimate cardiac action potential flow in the atria and detect AF sources.

The aim of this study is to explore the relation of extra PV sources to atrial anatomy in patients with persistent AF. We hypothesized that the characteristics and anatomical variations of LAA might correlate with active sources detected by EGF mapping in the LA, and it might predispose patients to a higher chance of AF recurrence.

## Materials and methods

### Study population

We analyzed cardiac computed tomography (CT) and EGF mapping data in patients who underwent *de novo* and redo radiofrequency CA for persistent AF at the Erasmus Medical Center, Rotterdam, Netherlands, between October of 2018 and March of 2021. In this study, a pre-procedural cardiac CT was performed in all included patients for in-depth assessment of LA anatomy. We collected demographic, anthropometric, clinical, and procedural data from all patients using the electronic health records and analyzed them in accordance with the hospital institutional review board policies (MEC-2019-0023). All subjects gave informed consent.

### Cardiac computed tomography imaging

**FIGURE 1:**
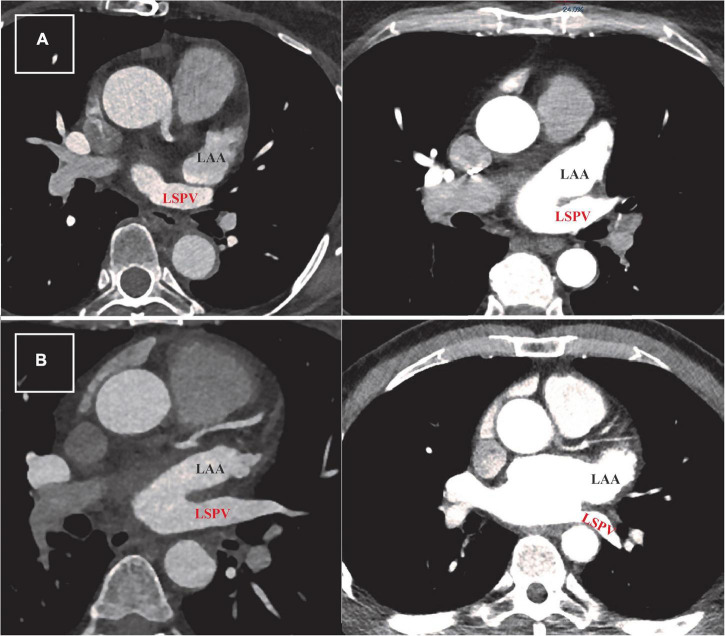
Defining abutting and non-abutting left atrial appendage–left superior pulmonary vein (LAA-LSPV). **(A)** Two examples of cardiac computed tomography (CT) scans from patients with abutting LAA (written in black) and LSPV (written in red). The measured distance between LAA and LSPV in these cases was less than 2 mm. **(B)** Two examples of CT scans from patients with persistent atrial fibrillation showing different trajectory of non-abutting LAA and LSPV. The measured distance between these two structures was more than 2 mm in both patients.

Our standard scan acquisition protocol consists of a high-pitch acquisition in the arterial phase covering at least the LA and a delayed phase high-pitch acquisition of at least the LAA. CT angiography examinations were performed with a 384-slice scanner (SIEMENS SOMATOM Force, Siemens Healthlineers) with prospective electrocardiogram (ECG)-triggered axial acquisition mode. For cardiac CT, 80–100 kV with 200–300°mAs tube current was used based on patient anthropometrics. Image acquisition was performed with 2 mm × 192 mm × 0.6 mm detector collimation and 0.25 s gantry rotation time. Heart rate control medication was not routinely administered prior to scanning. Contrast material was injected with a flow rate of 4.5–5.5 ml/s through antecubital vein access *via* 18-gauge peripheral IV using a four-phasic protocol. Bolus tracking in the LA was used to obtain the proper scan timing. CT datasets were reconstructed with 0.6-mm slice thickness with 0.4 mm increments. We examined LAA morphology classification, the approximate LAA length, diameters of the LAA ostium (axial, coronal, and sagittal plane), LAA shape, and relationship of the LAA to the left superior pulmonary vein (LSPV). The spatial relationship between LAA and LSPV was determined using two-dimensional axial CT images. Abutting LAA–LSPV was defined as cases when the LSPV touched the posterior aspect of LAA, and the maximal distance between the two structures was less than 2 mm (Figure 1A). Those cases where the distance between LAA and LSPV was more than 2 mm were defined as non-abutting LAA-LSPV (Figure 1B). CT scans were examined, and measurements were made by two-blinded observers (NS and JS).

### Electrophysiology procedure and catheter ablation

Antiarrhythmic drug therapy was not interrupted for patients prior to CA procedures. A decapolar catheter was advanced into the coronary sinus (CS), and AF was induced with decremental atrial burst pacing in all cases if the patient arrived in the electrophysiology lab in sinus rhythm. To reach activated clotting time (ACT) > 300 s before the introduction of the basket catheter, intravenous heparin was administered for every patient. Sustained AF of more than 5-min duration was recorded using a 64-pole basket catheter (FIRMap, Abbott, Abbott Park, IL, USA), which was introduced through an 8.5 Fr SL1 sheath in the RA and later in the LA. In redo cases, isolation of the PVs was checked and re-PVI was completed whenever necessary. The basket catheter was introduced into the LA and EGF mapping was performed. Using the EGF mapping software (Ablamap^®^, Ablacon, Inc., Wheat Ridge, CO, USA), clinically relevant active AF sources above the threshold (see below) were identified and then ablated using a 3.5 mm irrigated-tip catheter (Navistar^®^ RMT ThermoCool^®^, Biosense Webster, Irvine, CA, USA). Radiofrequency energy was applied with the following power settings: 25–50 W, temperature limit 43°C, flow rate 17–30 ml/min, using the Stereotaxis remote magnetic navigation system (Stereotaxis, St. Louis, MO, USA). In some cases, an extensive ablation at the LAA level was necessary.

### Electrographic flow mapping

The EGF mapping is a novel method used to estimate cardiac action potential flow in the atria that can detect AF sources in patients with persistent AF. EGF maps are generated from unipolar EGMs recorded from a 64-electrode basket catheter over 1 min. The software pre-processes these unipolar electrograms to remove far-field artifacts and normalizes the signals before they undergo flow analysis. The electrical potential profile between the electrode positions at a given point in time is estimated using Green’s algorithm assuming micro-electro-neutrality and undisturbed spreading of electrical fields. Using Horn-Schunk flow estimation, these Green’s interpolation frames taken every 19 ms are assembled to determine the spatial voltage gradient compared with the temporal voltage gradients derived from each two subsequent frames. The flow vector fields generated by the Horn–Schunk algorithm are analyzed to identify singularities where the flow vector angles around a point cover 360 degrees. These singularities are then evaluated for the divergence of the flow vector patterns to determine whether the singularity represents an active (divergent or centrifugal flow vectors) or passive (convergent or centripetal flow vectors) source. Because these origins of electrographic flow often occur repeatedly despite the stochastic variability of the flow fields in AF, this repetitive behavior can be integrated over time and is displayed in the EGF summary map. On EGF summary maps active sources appear redder the higher their rate of detection or prevalence and bluer the lower their prevalence. Correspondingly, passive rotors appear whiter the higher their prevalence and grayer the lower their prevalence. The radius of the source depends on the velocity detected around the singularity. The percentage of time a source can be detected at its highest intensity in a 19 ms segment is defined as source activity. Variability of the leading source is determined as the percentage of the surface of the total recording area in electrode distance units necessary to cover 80% of the source activity.

Patients with individual stable sources with a source activity above threshold (leading source > 26%) were classified as having an S-Type EGF signature with source-dependent AF. Patients with no stable active source pattern and no leading source with a source activity above threshold were characterized having a C-Type EGF signature consistent with source-independent AF. The classification of patient EGF signatures into S-Type and C-Type has been previously published (7). EGF source parameters were compared between subgroups of *de novo* and redo-PVI patients.

### Follow-up

Routine follow-up visits were scheduled at the outpatient clinic of our department for all patients 3, 6, and 12 months after the procedures. A total of 24-h Holter recordings were employed during these visits for documentation of recurrent arrhythmias. For long-term follow-up, patient records were analyzed. We compared echocardiography data between patients with AF recurrence and no AF recurrence, and between patients with AT recurrence and no AT recurrence. Outcome data were compared between subgroups of *de novo* and redo-PVI patients.

### Statistical analysis

Continuous variables are expressed as mean ± SD or median and quartiles. Categorical data are shown as percentages. We used the independent-samples *T* test and Pearson chi-Square to compare data between our groups. To measure inter-observer reliability, we used Cohen’s original Kappa analysis. Statistical analysis was performed using SPSS Statistics for Windows, Version 25.0.

## Results

### Demographic and baseline clinical data

Thirty patients were included in this study (mean age 62.3 ± 6.7 years). Three patients had concomitant atrial tachycardia/atrial flutter. In 23 patients, we found an S-Type EGF signature (77%), and in seven patients, we found a C-Type EGF signature (23%). We identified 10 patients with leading AF source near the LA ridge (structure between the left pulmonary veins ostia and the orifice of the LAA). The remaining 20 patients did not have a leading LA source near the ridge; six patients had a source at the LA roof; four patients had a source originating from the RA; two patients had a source in the septum; and two patients had a source originating inside the LAA. In five patients, we found an AF source localizing to the following areas: anterior wall, below LIPV, posterior wall, septum, near RSPV, and the infero-posterior wall. In one patient, we found no active AF source. Based on the EGF results, we created two patient groups: (1) patients with leading LA source near the ridge (*n* = 10); and (2) patients with no leading LA source near ridge (*n* = 20). Baseline patient demographics are summarized in Table 1. Catheter ablation was performed in 29 patients. Procedural data are summarized in Table 2. We further created two subgroups of patients: (a) patients who underwent prior PVI (*n* = 18), and (b) patients without prior PVI (*n* = 12). In subgroup (a), 13 patients (72.2%) had leading source near the LAA ridge; in subgroup (b), eight patients (66.6%) had leading source near the ridge (*p* = 0.46). In subgroup (a), 15 patients (83.3%) had an S-Type EGF signature; in subgroup (b), eight patients (66.6%) had S-Type EGF signature (*p* = 0.39).

**TABLE 1:** Demographic and main clinical data.

	Source near ridge	No source near ridge	*P*-value
Number of patients, n	10	20	
Age (years)	64.7 ± 8.4	61.3 ± 5.7	0.96
LVEF (%)	53.3 ± 10.1	52.6 ± 7.9	0.84
AF duration (years)	6.0 ± 3.8	7.4 ± 6.6	0.11
Prior PVI, n (%)	5 (50%)	13 (65%)	0.34
Hypertension, n (%)	7 (70%)	10 (50%)	0.25
Hyperlipidemia, n (%)	2 (20%)	4 (20%)	0.69
Diabetes, n (%)	3 (30%)	1 (5%)	0.09
Sleep apnea, n (%)	0 (0%)	2 (10%)	0.46
Ischemic heart disease, n (%)	2 (2%)	0 (0%)	0.10
Dilated cardiomyopathy, n (%)	0 (5%)	2 (10%)	0.43
CHA_2_DS_2_-VASc-score	2.3 ± 1.1	1.6 ± 1.4	0.92

AF, atrial fibrillation; LVEF, left ventricular ejection fraction; PVI, pulmonary vein isolation.

**TABLE 2:** Procedural data.

	Leading source near ridge	No leading source near ridge	*P*-value
Application number	33.0 (18.8–47.7)	26.5 (16.0–38.0)	0.21
Ablation time (s)	2206.8 ± 1250.2	1740.6 ± 734.5	0.21
Procedure duration (min)	215.8 ± 38.4	171.2 ± 45.76	0.01
Fluoroscopy dose (mGy)	190.5 (120.0–505.5)	243.5 (134.5–319.0)	1.00
DAP (mGy/cm^2^)	20118.2 (14434.5–50660.4)	20157.2 (12791.0–29809.7)	0.30

DAP, dose area product.

### Morphological and electrographic flow data

**FIGURE 2:**
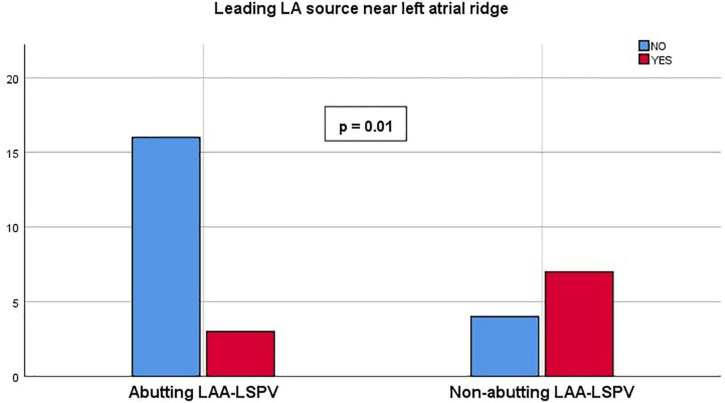
Leading left atrium (LA) source near left atrial ridge. Chart representing the presence of leading source near left atrial ridge compared between abutting and non-abutting left atrial appendage–left superior pulmonary vein (LAA-LSPV) patient groups.

The mean LAA length from the ostial plane to the LAA apex (primary lobe) was 42.1 ± 9.2 mm. Diameters of the LAA ostium measured in the axial, coronal and sagittal planes were 21.4 ± 4.3, 20.2 ± 2.7, and 20.0 ± 3.8 mm. There was no correlation found between LAA dimensions and leading AF sources near the ridge (*p* = 0.80). We identified four patients (13.3%) with chicken wing LAA shape, 22 patients (73.3%) with windsock, two patients (6.7%) with cauliflower, and two patients (6.7%) with cactus-shaped LAA. There was no correlation found between LAA shape and leading AF source near the ridge (*p* = 0.69). When analyzing the spatial relationship between LAA and LSPV, we identified 19 patients with abutting LAA-LSPV, and 11 patients with non-abutting LAA-LSPV. Three out of 19 patients presented with the leading AF source near the ridge in the abutting patient group, 7 out of 11 patients presented with the leading AF source near the ridge in the non-abutting patient group (*p* = 0.01) (Figure 2). In 24 patients, the leading source corresponded with the anatomical relation of LAA to LSPV on CT images described by our observers. LA data are summarized in Table 3 and EGF data are summarized in Table 4.

**TABLE 3:** Left atrium data.

	Source near ridge	No source near ridge	*P*-value
LA diameter (mm)	48.4 ± 8.6	47.8 ± 6.6	0.84
LAVI (mL/m^2^)	57.3 ± 8.5	44.6 ± 11.7	0.06
LAA length (mm)	41.5 ± 6.9	42.5 ± 10.6	0.78
LAA ostium diam. horizontal (mm)	21.3 ± 4.9	21.7 ± 4.1	0.80
LAA ostium diam. coronal	19.1 ± 2.6	20.8 ± 2.7	0.13
LAA ostium diam. sagittal	18.9 ± 3.7	20.6 ± 3.9	0.28
Ao. Ascendens (mm)	37.1 ± 7.9	37.1 ± 5.1	0.98
Ao. Sinus (mm)	35.0 ± 1.0	38.0 ± 7.2	0.51
TAPSE	21.4 ± 6.7	22.9 ± 6.2	0.57

LA, left atrium; LAVI, left atrial volume index; LAA, left atrial appendage; Ao, aorta; TAPSE, tricuspid annular plane systolic excursion.

**TABLE 4:** Electrographic flow data.

	Abutting	Non-abutting	*P*-value
Highest single activity value	35.9 ± 13.5	37.8 ± 16.0	0.70
Left atrial active source	16/19	10/11	0.53
Type S recordings	15/19	8/11	0.51
Leading LA source near ridge	3/19	7/11	0.01

LA, left atrium; EGF, electrographic flow.

Regarding the classification of abutting *vs*. non-abutting LAA-LSPV, the inter-observer reliability calculated with Cohen’s Kappa test was 0.92, showing a high level of agreement between the two observers.

### Follow-up data

Follow-up data show that 3 out of 30 patients presented with symptomatic AF recurrence during the 12-months follow-up period. All three patients had non-abutting LAA-LSPV and presented leading AF source near the ridge (AF recurrence in abutting *vs*. non-abutting LAA-LSPV patient group 0 *vs*. 3, *p* = 0.05). Patients with AF recurrence had larger LA diameters (56.7 *vs*. 46.2 mm, *p* = 0.01) and higher LAVI values (61.0 *vs*. 42.8 ml/m^2^, *p* = 0.01) than AF free patients. In subgroup (a), two patients (11.1%) had documented recurrence; and in subgroup (b), one patient (8.3%) had AF recurrence (*p* = 1.00). Six additional patients presented with atrial tachycardia during the 12-months follow-up period. In subgroup (a) four patients (22.2%), in subgroup (b) two patients (16.6%) had documented AT recurrence (*p* = 1.00). Comparing echocardiography data between the AT recurrence and AT free patient groups, we found that LAA ostium sagittal diameters were larger in the AT recurrence group (23.8 *vs*. 18.7 mm, *p* < 0.01). We found no differences in LAA length (43.7 ± 11.7 *vs*. 41.8 ± 9.0, *p* = 0.66), LA diameter (47.6 ± 3.5 *vs*. 48.1 ± 7.9, *p* = 0.89), LA volume (87.5 ± 29.2 *vs*. 96.5 ± 35.9, *p* = 0.66), LAVI (43.5 ± 14.8 *vs*. 47.9 ± 12.2, *p* = 0.63), and LVEF (50.0 ± 10.6 *vs*. 53.6 ± 7.9, *p* = 0.36) between AT recurrence and AT free patient groups. Two patients underwent a repeat procedure.

## Discussion

This is the first study to evaluate the correlation between anatomical characteristics of the LA (including LAA) and the presence of extra-PV sources in patients with persistent AF. We found that LA characteristics and LAA dimensions do not correlate with the presence of extra PV sources; however, the spatial relationship between the LAA and LSPV showed a significant correlation with the presence of sources in the LA. In patients with a non-abutting LAA-LSPV, EGF maps showed frequent AF sources near the LA ridge.

There appears to be a ceiling of efficacy achieved with PVI-alone for the treatment of persistent AF and as such, extra PV sources like the LAA have been proposed to play a role in the initiation and maintenance of AF. Examining 987 consecutive patients undergoing redo-CA for AF, 71% of which were non-paroxysmal, Di Biase et al., found that 27% of patients with intact PVI had to fire from the LAA, and of these patients, in 8.7% of the LAA was the only apparent source of AF (4). In a study conducted by Hocini et al. it is demonstrated that the LAA is an important source of localized reentrant atrial tachycardias after unsuccessful AF ablations in patients with persistent AF (8). Consistent with the literature, this research found that the LAA does play a role in source localization in patients with persistent AF.

Due to the complex embryological development of the LA, LAA, and pulmonary veins, the LAA may represent a potential conduction zone for arrhythmogenesis. Douglas et al., described that the PV walls are surrounded by extra-pericardially differentiated myocardial cells that become incorporated into the LA myocardium along with the PVs (9). However, the LAA consists of both endocardial and myocardial layers without the PV wall component, creating a discontinuity of myocardium such that the LAA may initiate AF similar to the PVs (9).

Small clinical studies have been published associating the morphological structure of the LAA with AF recurrence after radiofrequency ablation and cryoablation (10–14). A more recently published study evaluated the anatomical relationship between LAA and LSPV in paroxysmal AF patients in a large cohort. It was shown that the spatial relationship of the LAA and LSPV has an impact on the efficacy of AF ablation (15, 16). Our current findings put previous data into perspective. Although our study was powered as a morphology study and for outcome it is underpowered, it is still surprising to appreciate the difference in AF recurrences during the 12 months follow-up period. The number of recurrences in patients presenting leading AF source near ridge *vs*. in patients with no leading AF source near ridge is different, suggesting that recurrences appear more likely in patients with non-abutting LAA-LSPV, with larger LA diameters, and higher LAVI values. Interestingly enough, we also found that AT recurrences are more frequently observed in patients with larger LAA ostial sagittal diameter. Based on this, we hypothesize that LA anatomical variations do play a role in AF source localization and also in arrhythmia recurrence.

There are several approaches based on phase mapping and activation mapping used for the identification of AF sources (17). However, software algorithms based on phase and/or activation mapping suffer significant technical limitations, such as poor spatiotemporal resolution, lack of map reproducibility over time, creation of false positives and epiphenomena, and the inability to differentiate between active and passive AF sources (18, 19). Modern mapping systems with ultra-high density mapping tools offer other possible approaches, such as stochastic trajectory analysis of ranked signals (STAR mapping), to guide AF ablation with promising initial results (20). These novel concepts contribute to the improvement of invasive electrophysiology procedures by shortening procedure times and decreasing fluoroscopy use. Utilizing all available technical possibilities and tailoring non-fluoroscopic imaging tools to the electrophysiology procedures, X-ray exposure can be minimized (21). The radiation dose and risk awareness during electrophysiology procedures is fundamental today in the risk-benefits assessment (22).

**FIGURE 3:**
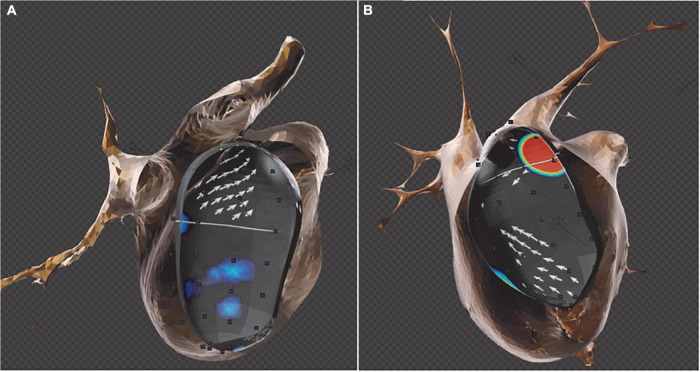
Merged computed tomography (CT) and electrographic flow (EGF) map reconstructions. Sources detected by the EGF software are projected on the CT surface to demonstrate the exact anatomical location within the cardiac chamber. **(A)** EGF map reconstruction shows electric flow emerging from the left inferior pulmonary vein (LIPV) (see white arrows). **(B)** EGF reconstructions identify an extra-PV source emerging from the ridge between left atrial appendage–left superior pulmonary vein (LAA-LSPV) (orange patch).

The EGF mapping is the first technology to discriminate between active and passive rotors during endocardial mapping (23). EGF mapping enables the spatial and temporal reconstruction of electrographic potentials derived from endocardial unipolar electrogram data (24). Source detection with EGF mapping uses an optical flow algorithm that provides better spatial resolution than the basket grid constant used to record the signals (Figure 3). The inter-procedural reproducibility and spatiotemporal stability of EGF maps and EGF-identified sources suggest that signal acquisition using a basket catheter is sufficient for the localization of AF sources (25). Its ability to classify AF as being source-dependent (S-type EGF signature) versus source-independent (C-type EGF signature) may be clinically useful for patient stratification and ablation strategy planning (7). Using this technology, non-PV sources (S-type signatures) can be detected and targeted with ablation to improve clinical outcomes.

Our results demonstrate a trend toward less efficient CA procedures (longer procedures, more fluoroscopy, and RF applications) in patients with leading source near ridge. This can be explained by the necessity of more excessive ablation of AF sources identified in the ridge area.

The EGF mapping offers a new perspective in active source detection in persistent AF. With this technology, we are now able to identify and target extra-PV sources, which together with the standard PVI technique may result in improved ablation outcomes, particularly for patients with persistent AF. EGF mapping opens new possibilities in the assessment of unexplored correlations between extra-PV sources and LA characteristics. Although the BELIEF randomized study suggested that empirical electrical isolation of the LAA improved ablation outcomes for long-standing persistent AF patients, electrical isolation of the LAA poses a potential thromboembolic risk, requiring systemic anticoagulation (6). The identification of focal extra-PV sources of AF along the LA ridge may allow for targeted ablation of these triggers without the need for empiric LAA isolation. Further large-scale, prospective studies are required.

### Study limitations

This was an exploratory study of patients who prospectively underwent EGF mapping and ablation of EGF-identified sources with a relatively small sample size and has important limitations. Although, this can be attributed to the fact that the above-mentioned technology is commercially not yet available. The use of a basket catheter to record intracardiac signals raises the possibility of non-uniform endocardial contact, basket spline deformation such as bunching and splaying, and incomplete atrial coverage that may affect the quality of signal acquisition. The most accurate follow-up includes loop recorder implantation for possible asymptomatic recurrence detection, quality of life evaluation, and long-term mortality and morbidity assessment (26). Although patients were followed clinically including ECG and Holter monitoring, asymptomatic AF recurrences may have been missed resulting in an underestimation of the overall recurrence rate during follow-up.

### Future perspectives

Electrographic flow (EGF) is a novel technology especially designed to account for spatially incoherent arrhythmias like AF. Our center is one of the five centers in the EU where this technology is available. We strongly believe that if implemented in everyday clinical practice, this novel technology will help to better understand the underlying mechanisms of AF, and offer an optimal treatment option for patients suffering from persistent AF. As a future perspective, combining 3D CT images and EGF mapping data with VR (virtual reality) we would be able to achieve a better visual inspection of anatomy, and extra-PV source identification.

## Conclusion

The presence of EGF-identified extra-PV sources near the LA ridge correlates with the anatomical relation of the LAA to the LSPV in patients with persistent AF.

## Data availability statement

The data that support the findings of this study are available from the corresponding author (TS-T) upon reasonable request.

## Ethics statement

This study was reviewed and approved by The Erasmus MC MERC. The patients/participants provided their written informed consent to participate in this study.

## Author contributions

TS-T and RG outlined and drafted the manuscript. JS and NS examined radiology data. MK and PR provided technical data and images for the manuscript. SW and RB provided the critical revision with insightful and constructive comments to improve the manuscript. All authors contributed to the article and approved the submitted version.
